# Beyond Anti-PD-1/PD-L1: Improving Immune Checkpoint Inhibitor Responses in Triple-Negative Breast Cancer

**DOI:** 10.3390/cancers16122189

**Published:** 2024-06-11

**Authors:** Kennady K. Bullock, Ann Richmond

**Affiliations:** Department of Pharmacology, School of Medicine, Vanderbilt University, Nashville, TN 37232, USA; kennady.k.bullock@vanderbilt.edu

**Keywords:** triple-negative breast cancer, immune checkpoint inhibition, PD-1, PD-L1, PI3K/AKT, RAS/MAPK/ERK

## Abstract

**Simple Summary:**

Triple-negative breast cancer (TNBC) is a challenging-to-treat subtype of breast cancer with limited treatment options beyond standard chemotherapy and surgery. The introduction of immune checkpoint inhibitor (ICI) therapy with anti-PD-1 for the treatment of TNBC represents a great advance in the field, yet few patients achieve long-lasting responses to therapy. The goal of this review is to discuss key efforts to improve ICI response rates in TNBC patients. The three conceptual strategies discussed include improving patient selection, identifying existing therapies that may enhance anti-PD-1 therapy, and identifying new immunotherapy strategies together outside of the PD-1 axis.

**Abstract:**

The introduction of anti-programmed cell death protein-1 (anti-PD-1) to the clinical management of triple-negative breast cancer (TNBC) represents a breakthrough for a disease whose treatment has long relied on the standards of chemotherapy and surgery. Nevertheless, few TNBC patients achieve a durable remission in response to anti-PD-1, and there is a need to develop strategies to maximize the potential benefit of immune checkpoint inhibition (ICI) for TNBC patients. In the present review, we discuss three conceptual strategies to improve ICI response rates in TNBC patients. The first effort involves improving patient selection. We discuss proposed biomarkers of response and resistance to anti-PD-1, concluding that an optimal biomarker will likely be multifaceted. The second effort involves identifying existing targeted therapies or chemotherapies that may synergize with ICI. In particular, we describe recent efforts to use inhibitors of the PI3K/AKT or RAS/MAPK/ERK pathways in combination with ICI. Third, considering the possibility that targeting the PD-1 axis is not the most promising strategy for TNBC treatment, we describe ongoing efforts to identify novel immunotherapy strategies.

## 1. Introduction

**Figure 1:**
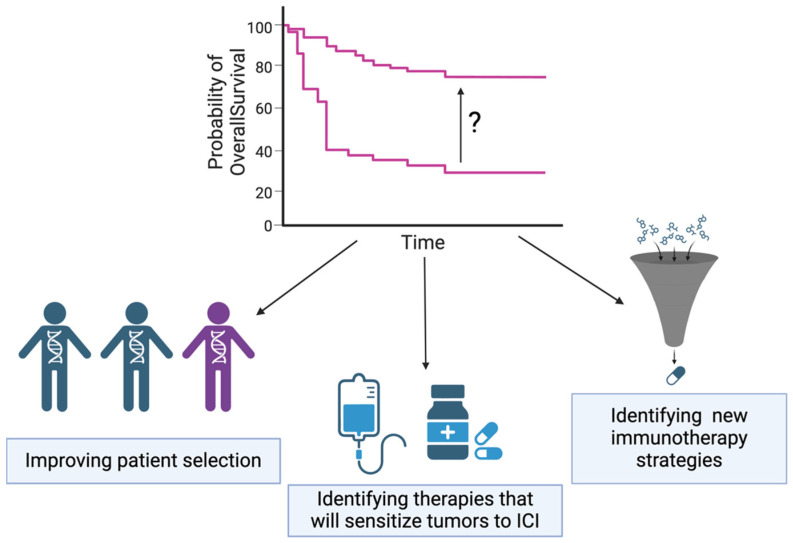
Conceptual strategies to improve TNBC response rates to ICI. Three strategies to improve ICI response rates in TNBC include improving patient selection, identifying therapies that will sensitize tumors to ICI, and identifying new immunotherapy strategies. Created with BioRender.com (accessed on 9 May 2024).

Breast cancer (BC) is the most common cancer among women worldwide. Early detection strategies and the development of hormone receptor-targeted therapeutics revolutionized breast cancer care, allowing women to sometimes survive decades with the disease. Between 1989 and 2020, the overall death rate from breast cancer decreased by 43%, and the current overall relative survival rates for women 5 years and 15 years after diagnosis are 91% and 80%, respectively [1]. The prognosis is affected by the stage of diagnosis as well as the tumor subtype. Triple-negative breast cancer (TNBC) is a particularly aggressive subtype of BC, accounting for about 10% of all BC cases in the US [1]. The improvements in patient outcomes for TNBC have not matched those of hormone receptor (HR) positive subtypes, and the five-year relative survival rate for TNBC is 77% [1]. TNBC is broadly characterized by a lack of human epidermal growth factor receptor 2 (HER2) amplification, a lack of estrogen receptor (ER) expression, and a lack of progesterone receptor (PR) expression. The lack of these pharmacologically targetable receptors limits therapeutic options for TNBC patients. Consequently, treatment relies heavily on standard chemotherapy and surgery. Recent advancements include the approval of poly-ADP ribose polymerase (PARP) inhibitors for breast cancer gene (BRCA)-mutant, HER2 negative BC, and the approval of the immune checkpoint inhibitor, anti-PD-1, in the neoadjuvant and adjuvant settings [2]. However, few BC patients exhibit a durable response to immune checkpoint inhibition (ICI), and there remains an urgent need to develop new treatment strategies for TNBC patients. In the present review, we will discuss efforts to improve immunotherapy response rates in BC, with a focus on TNBC. Ongoing efforts include improving patient selection to identify who would benefit most from existing ICI, identifying existing therapies that can be used in combination with ICI to boost response, and identifying new immunotherapy strategies together outside of the conventional anti-PD-1/programmed death-ligand 1 (PD-L1) approach (Figure 1).

## 2. TNBC Heterogeneity and Sub-Classifications

TNBC treatment is complicated by the heterogeneity that exists within this subtype. While all TNBCs are defined by a negative histologic score for HRs, some TNBCs express a low level of HER2. Such tumors receive a 1+ or 2+ HER2 score by immunohistochemistry (IHC) and thus fall into the newer classification of HER2-low TNBC [3]. A 3+ IHC score (greater than 10% positive cells) or amplification detected by in situ hybridization (ISH) is required for a tumor to receive a HER2+ classification [4]. A HER2-targeted antibody–drug conjugate, trastuzumab deruxtecan, is being investigated as a new treatment specifically for HER2-low BC [5]. Beyond histological classification, four tumor-specific molecular subtypes of TNBC are currently recognized: basal-like-1 (BL1), basal-like-2 (BL2), mesenchymal (M), and luminal androgen receptor (LAR) [6]. Ongoing work is describing which subtypes may respond best to which available therapies. For example, patients with the BL1 subtype are more likely to achieve a pathological complete response (pCR) after neoadjuvant chemotherapy (NAC) [6]. There is also heterogeneity within the immune cell profiles of TNBC tumors, which will be discussed in detail in subsequent sections. In a retrospective analysis of over 1000 pre- and post-treatment biopsies, high levels of tumor-infiltrating lymphocytes (TILs) were associated with a higher pCR after chemotherapy [7]. The association between TILs and pCR is particularly strong in TNBC tumors, and later studies confirmed TILs as a predictive factor of response to neoadjuvant therapy in TNBC [8]. High TILs are also associated with improved overall survival in TNBC, particularly in early-stage disease [9]. Compared to HR+ and HER2+ tumors, TNBC tumors are more likely to exhibit high TILs, and TNBC was the first breast cancer subtype considered as a candidate for immune checkpoint inhibitor trials.

## 3. Immunotherapy and Immune Checkpoint Inhibition

**Figure 2:**
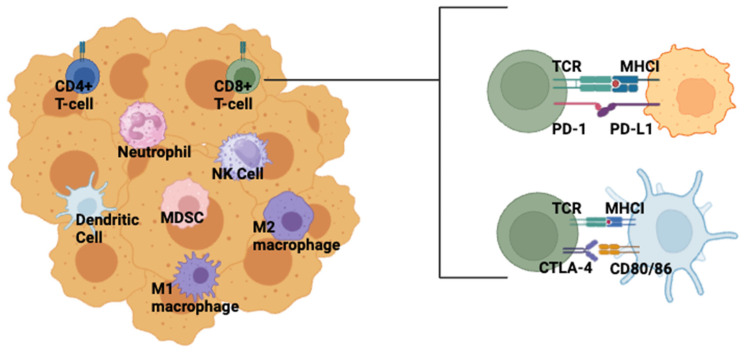
PD-1, PD-L1, and CTLA-4 in the TME. Schematic of examples of the diverse immune cells present in the TME and the mechanisms of action of the immune checkpoints PD-1, PD-L1, and CTL-4. PD-L1 is expressed by many cell types, including tumor cells (depicted), stromal cells, and macrophages [20]. CTLA-4 binds to CD80/86 expressed on antigen-presenting cells (depicted) [21]. Created with BioRender.com (accessed on 9 May 2024).

Tumors are a conglomeration of different cell types, each able to influence disease progression and response to therapy. Immune cells, including those of the lymphoid and myeloid lineages, are key components of the tumor microenvironment (TME) (Figure 2), affecting disease trajectory and response to therapies. Recognizing the importance of the TME, Hanahan and Weinberg updated the Hallmarks of Cancer paradigm in 2011 to include the avoidance of immune destruction as a key property of tumor biology [10]. The cancer immunoediting model can better conceptualize the role of immune cells in shaping tumor resistance to anti-tumor immunity. According to the model put forth by Schreiber, the immune system constantly surveils the tissue microenvironment for the early development of cancerous cells, and those cells must progress through three stages—elimination, equilibrium, and escape—to form a malignant tumor [11]. In the elimination phase, cells of the innate immune system, including macrophages, natural killer (NK) cells, dendritic cells, and innate T-cells, including natural killer T-cells (NKTs) and y*δ*T-cells recognize and eradicate aberrant cells [11]. Exactly how these cells are recognized is an active area of research; however, some theories include inflammatory signals released in response to enhanced angiogenesis [12] or the presence of neoantigens [13]. A response from the adaptive arm of the immune system involving tumor-specific CD4+ and CD8+ T cells may also occur. In the second stage, equilibrium, the tumor cells are contained in a dynamic equilibrium whereby the immune response prevents further tumor growth but does not eradicate the existing cells [11]. The surviving cells continue to acquire mutations, eventually entering the third stage—escape. In this stage, the tumor cells continue to grow unchecked by the immune response, resulting in the formation of a clinically apparent tumor. The field’s knowledge of the complex relationships between the immune system and tumor development continues to evolve, and understanding the role of the immune system in cancer changes our understanding of how to best treat cancer. 

The basic strategy behind immunotherapy is to leverage the body’s immune system to fight its own cancer, much like it would fight any other pathogen. The earliest immunotherapy effort is often credited to William Coley, who used Coley’s toxin—a bacterial mixture of Streptococcus pyogenes and Serratia marcescens—for the treatment of soft tissue sarcomas in the 1800s [14]. While some of Coley’s patients experienced tumor regressions, the field’s understanding of immunology was not yet advanced enough to explain the mechanism behind the responses, and Coley’s toxin was abandoned [15]. Immunotherapy strategies have progressed as our understanding of the basic biology of the immune system has improved, and immune checkpoint inhibitors are now key players in the current clinical treatment of cancer. Immune checkpoint molecules exist to negatively regulate the immune response and protect the body from autoimmunity. In the context of cancer, the engagement of such checkpoint proteins can prevent an immune response from occurring against a tumor cell. Checkpoint proteins can be blocked with antibodies to postpone this downregulation of the immune response, known as immune cell exhaustion. Programmed cell death protein 1 (PD-1) and cytotoxic T-lymphocyte-associated protein 4 (CTLA-4) are two checkpoint molecules targeted by immunotherapies in recent years, and the 2018 Nobel Prize in Physiology or Medicine was awarded to Tasuku Honjo and James Allison for their discoveries and work with these checkpoint molecules [16].

PD-1 is expressed on the surface of T-cells after T-cell receptor (TCR) stimulation [17]. PD-1 binding to ligand, either programmed death ligand 1 or 2 (PD-L1 or PD-L2), initiates an intracellular cascade involving the recruitment of the phosphatases Src homology-2 domain-containing phosphatases 1 and 2 (SHP1 and SHP2), which inactivate intracellular downstream effectors of T-cell activation, such as the transcription of key genes required for T-cell proliferation and activation, including activator protein-1 (AP-1) and nuclear factor of activated T-cells (NFAT). This cascade eventually leads to either T-cell exhaustion or apoptosis [18,19]. PD-L1 is expressed by antigen-presenting cells and can be upregulated by tumor cells as an immune evasion mechanism [20]. Either PD-1 or PD-L1 can be blocked with therapeutic antibodies. Like PD-1, CTLA-4 expression is induced on the surface of T cells after activation by antigen recognition. Unlike PD-1, CTLA-4 is constitutively expressed at low levels on regulatory T-cells (CD4+CD25+ cells). CTLA-4 structurally resembles CD28, the receptor necessary for co-stimulation of the TCR. Both CTLA-4 and CD28 can bind CD80 and CD86, which are expressed by antigen-presenting cells. When CTLA-4 binds to CD80/CD86, a similar intracellular cascade occurs as in the PD-1/PD-L1 axis, whereby phosphatases are recruited that inactivate signaling necessary for transcription of genes involved in T-cell proliferation and activation [21]. CTLA-4 additionally internalizes ligands upon binding, preventing CD28 from binding CD80/CD86. Consequently, the T-cell does not receive the necessary co-stimulatory signal to respond to antigen [22]. The PD-1/PD-L1 axis and CTLA-4/CD80/86 axis both act as negative regulatory safeguards of the immune system, and mice genetically deficient in either CTLA-4 or PD-1 exhibit autoimmune phenotypes [23,24].

## 4. Clinical Status of ICI in TNBC

Anti-PD-1 is currently approved by the Food and Drug Administration (FDA) for use in TNBC as the result of several key clinical trials. The phase III IMpassion130 trial tested atezolizumab (anti-PD-L1) in combination with nanoparticle albumin-bound paclitaxel (nab-PTX) as a first-line treatment for locally unresectable or metastatic TNBC. Progression-free survival (PFS) in the anti-PD-L1 + nab-PTX group was 7.2 months, while PFS in the nab-PTX + placebo group was 5.5 months. Post hoc analysis showed an improvement in overall survival (OS) in patients with PD-L1+ tumors (21.3 vs. 17.6 months) [25]. While the approximately two-month improvement in PFS was modest, there are few therapies available for the late-stage patients represented by the trial, and the IMpassion130 results led to the accelerated regulatory approval of anti-PD-L1 + nab-PTX for metastatic TNBC in 2019. Unfortunately, in the subsequent confirmatory IMpassion131 trial, atezolizumab failed to improve PFS or OS, even in the PD-L1+ subgroup [25], and the accelerated regulatory approval was revoked. 

Concurrent to the IMpassion trials, the phase III KEYNOTE-355 trial tested pembrolizumab (anti-PD1) in combination with physician’s choice chemotherapy (paclitaxel (PTX), nab-PTX, or carboplatin + gemcitabine) as a first-line treatment for metastatic TNBC. The addition of anti-PD-1 to chemotherapy improved PFS (9.7 vs. 5.6 months) and OS (23.0 vs. 16.1 months) in patients with PD-L1+ tumors [26,27]. KEYNOTE-355 led to the regulatory approval of pembrolizumab plus chemotherapy for use in PD-L1+ metastatic TNBC in the United States in 2020. Toripalimab, an anti-PD-1 antibody first approved for use in metastatic melanoma in China in 2018, is also being investigated in combination with nab-PTX for stage IV TNBC in the TORCHLIGHT phase III trial (NCT04085276). Interim analysis shows that the addition of toripalimab significantly improves median PFS (8.4 vs. 5.6 months) and median overall survival (32.8 vs. 19.5 months) compared to nab-PTX alone in PD-L1+ patients [28].

Several clinical trials also investigated anti-PD1 and anti-PD-L1 in the neoadjuvant setting for early-stage TNBC. Phase II GeparNUEVO found that the addition of durvalumab (anti-PD-L1) to anthracycline–taxane-based neoadjuvant chemotherapy trended toward improving pCR compared to chemotherapy alone, especially in tumors with high stromal tumor-infiltrating lymphocytes (sTILs) [29]. In the phase II NeoPACT trial, the addition of pembrolizumab to carboplatin plus docetaxel yielded a pCR of 58%, suggesting the benefit of an anthracycline-free-based treatment strategy [30]. In the phase III IMpassion031 trial, the addition of atezolizumab (anti-PD-L1) to neoadjuvant nab-PTX/doxorubicin/cyclophosphamide significantly improved pCR (58% vs. 41%) [31]. Finally, in the Phase III KEYNOTE-522 trial, the addition of pembrolizumab to neoadjuvant PTX/carboplatin improved pCR (64.8% vs. 51.2%) [32], leading to the 2021 regulatory approval of pembrolizumab as a neoadjuvant treatment for early-stage TNBC, regardless of PD-L1 status. Results from a later interim analysis of KEYNOTE-522 show that pembrolizumab plus neoadjuvant chemotherapy also significantly extends event-free survival (EFS) compared to neoadjuvant chemotherapy alone, with an estimated EFS at 36 months of 84.5% compared to 76.8% [33]. Largely because of the KEYNOTE-522 trial, pembrolizumab plus chemotherapy (paclitaxel, carboplatin, doxorubicin, and cyclophosphamide) is the standard of care for most stage II and III TNBC patients, and this 5-drug combination is referred to as the K522 regimen [34]. The K522 regimen represents an intensive therapy, and there is great interest in de-escalation strategies to minimize the amount of chemotherapy that can be used in combination with anti-PD-1 while still maintaining clinical benefit [30]. While anti-PD1 is now used in the clinic for TNBC, few patients achieve durable remission. The improvements in PFS and EFS are especially short when we consider the benefits ICI has had on other tumor types, such as melanoma, where the PFS is on the scale of years rather than months [35,36]. 

## 5. Improving Patient Selection

Given the diversity within the TNBC designation, efforts to further improve patient selection to minimize potential adverse effects and maximize the potential benefit of immune checkpoint inhibitors are essential. Several response criteria have been proposed, but there is not currently a reliable biomarker to guide treatment decisions. PD-L1 expression was initially proposed as a patient selection marker. PD-L1 expression is correlated with response to anti-PD1 therapy in the metastatic setting [26,27]; however, response in the early disease setting is independent of PD-L1 status [32]. PD-L1 is a problematic biomarker, as detection methods and criteria for positivity can vary between different hospitals and laboratories. Different PD-L1 antibodies and different scoring systems are used for IHC analysis [37]. Furthermore, PD-L1 can be expressed by multiple cell types in the TME, including tumor cells, stromal cells, macrophages, and TILs [20,38]. Expression of PD-L1 in CD68+ macrophages is associated with higher rates of pCR in response to durvalumab (anti-PD-L1) plus chemotherapy in pretreatment core needle biopsies from TNBC patients participating in phase I/II clinical trial (NCT02489448) of neoadjuvant durvalumab in combination with nab-PTX and dose-dense doxorubicin and cyclophosphamide (ddAC) [39]. There is a push to incorporate cell type expression and localization of PD-L1 positivity into the current PD-L1+ scoring system, as these are important aspects of histologically assessing a clinical specimen [37]. In recent clinical trials, PD-L1 status has been described in terms of tumor cell versus immune cell positivity. In the phase II GeparNUEVO trial, PD-L1 expression was assessed as both the percentage of positive tumor cells (PD-L1-TC) and the percentage of positive immune cells (PD-L1-IC) [29]. Interestingly, PD-L1-IC correlated with increased pCR in the placebo plus chemotherapy group rather than the durvalumab plus chemotherapy group [29]. PD-L1 is not the isolated biomarker for ICI response that the field initially hoped for, but cell-type-specific PD-L1 positivity may be a useful piece in the overall diagnostic picture. While efforts are underway to standardize and improve PD-L1 detection and scoring systems, anti-PD1 therapy is currently FDA-approved for TNBC, regardless of PD-L1 status.

### Immune Cell Signatures

High levels of TILs in pre-treatment biopsies are associated with a greater likelihood of pCR to neoadjuvant chemotherapy in TNBC [7], and immune cell infiltrate is also being investigated as a potential marker of response to ICI. Initial immunotherapy efforts in breast cancer focused on triple-negative rather than HR+ tumors because TNBC is thought to have a higher immune cell infiltrate. However, there is a wide range of immune cell profiles just within the TNBC designation. Tumors can broadly be described as either “hot” or “cold” tumors, meaning the presence or absence of anti-tumor immune cells, respectively. Cytotoxic CD8+ T-cells, M1-like macrophages, and NK cells are considered anti-tumor immune cells, whereas regulatory T-cells (Tregs) and M2-like macrophages are examples of immunosuppressive cells that may be found in a “cold” tumor [40]. The immune cell milieu of a tumor can change, however, both in response to prior treatment and to disease progression. For example, analysis of paired samples from TNBC primary and metastatic sites shows downregulation of immunomodulatory gene signatures in metastatic samples [41]. 

Gene expression studies of responding and non-responding tumors can provide insight into immune-based biomarkers. For example, RNA-sequencing data from tumors of TNBC patients who received neoadjuvant durvalumab in combination with chemotherapy were used to define a 27-gene immuno-oncology (IO) signature that predicted pCR [42]. Tumors positive for the IO signature also had higher CD8+ T-cells predicted by CIBERSORT and higher CD4+ T-cells predicted by TIMER. CYBERSORT and TIMER are two computational programs that predict the abundance of specific cell types based on gene expression data [43,44]. Combining the IO signature with PD-L1 IHC analysis further strengthened the pCR predictive capabilities of the proposed model [42]. Another group, focusing on myeloid components, identified a neutrophil-enriched subtype (NES), defined by the accumulation of immunosuppressive neutrophils and granulocytic myeloid-derived suppressor cells, that is associated with resistance to ICI therapy in TNBC [45]. Recently, a method called T-cell InteractPrint was developed, which calculates predicted interactions between heterogenous epithelial cancer cells and CD8+ T-cells to predict response to anti-PD-1 based on single-cell ribonucleic acid sequencing (scRNA-seq) from TNBC tumors [46]. In the datasets tested, the T cell InteractPrint score was a better predictor of response to anti-PD-1 in the TNBC early disease setting than PD-L1 status [46]. Furthermore, the BioKey window-of-opportunity study (NCT03197389) was designed to generate a single-cell map of the intratumoral changes that occur in breast cancer in response to anti-PD1 therapy [47]. Treatment-naive patients received either one dose of anti-PD1 before surgery or 20-24 weeks of chemotherapy plus one dose of anti-PD1 before surgery. Pre-treatment and on-treatment biopsies were collected and subjected to scRNA-seq and single-cell t-cell receptor sequencing (scTCR-seq). Clonal expansion of cytotoxic CD8+ T-cells was observed after anti-PD1 treatment, and the following cell types were associated with T-cell expansion: Major histocompatibility complex I/II (MHCI/II)-expressing cancer cells, PD-L1+ dendritic cells, and CCR2+/MMP9+ macrophages [47]. scRNA-seq and scTCR-seq were also used to assess immune cell changes in the primary tumor and peripheral blood of TNBC patients treated with PTX and anti-PD-L1 [48]. Baseline B-cell populations that highly expressed genes related to antigen processing/presentation and T-cell activation were most predictive of response to PTX+anti-PD-L1, while responsive tumors exhibited expansion of tumor-reactive T-cell populations, including CXCL13+ CD4+ and CXCL13+ CD8+ T-cells [48]. Efforts to define gene signatures associated with response are resulting in the incorporation of gene expression as a secondary outcome in clinical trials. For example, in the phase II GeparNUEVO study of neoadjuvant ICI + chemotherapy in TNBC, RNA-seq was performed on pre-treatment biopsies to analyze an immune gene expression profile (GEP) of 10 previously defined genes, and GEP was independently associated with and predicted pCR [49].

While sequencing data can predict the abundance of particular cell types, the spatial localization, not just the presence or absence of immune cells, is important. Tumors can be divided into three phenotypes that describe the localization of immune cells: inflamed, immune-excluded, and immune-desert [50]. The inflamed phenotype describes tumors with high levels of TILs and high levels of inflammatory cytokines that favor T-cell activation and anti-tumor immune responses. Inflamed tumors are the most likely to respond to ICI. In the immune-excluded phenotype, anti-tumor immune cells are present, but they are restricted to the surrounding stroma, unable to infiltrate the tumor. The immune-desert phenotype describes tumors lacking CD8+ T-cells both in the tumor interior and surrounding stroma. Consequently, immune-desert tumors are the least likely to respond to ICI. Histological analysis and spatial transcriptomics can provide nuanced information on cell type localization. Histological analysis of TILs can be achieved with a simple hematoxylin and eosin (H&E) stain, and machine learning algorithms are being developed to standardize tissue scoring [37,51]. High TILs, as measured by histological analysis, were significantly associated with the pCR rate in patient samples from the KEYNOTE-522 trial of neoadjuvant pembrolizumab in combination with chemotherapy [34]. Beyond existing histology pipelines, work is being conducted to combine histological analysis and spatial transcriptomics to further subdivide TNBC immunophenotypes. In one study, treatment-naive TNBC biopsies were classified based on IHC staining for CD8+ cells with low infiltration into the tumor core (corCD8lo) and high infiltration into the tumor core (corCD8hi) [52]. The corCD8lo was further subdivided into the immune desert (ID) or margin-restricted (MR), while the corCD8hi was further divided into stroma-restricted (SR) or fully inflamed (FI). Each spatially distinct phenotype also expressed unique gene signatures and was correlated to response to therapy when applied to independent data sets. For example, the FI subtype expressed a type I IFN gene signature, GzmB+CD8+ T-cells, and CD68+CD206- macrophages, while the SR subtype expressed elevated levels of IL-17+ *γδ*T-cells. Importantly, while both the FI and SR subtypes exhibit CD8 infiltration into the tumor core, only the FI subtype is correlated with an increased response to ICI [52]. Another study used imaging mass cytometry to explore the protein expression profiles of TNBC tumors before and after neoadjuvant ICI, finding that proliferating CD8+TFC1+ T-cells and proliferating MHCII+ cancer cells were most predictive of response to ICI [53]. The most effective predictor of response will likely be a composite biomarker encompassing the multiple characteristics discussed above. A further challenge after identifying a biomarker will be standardizing methods of detection and ensuring testing accessibility to all relevant clinical populations. 

## 6. Identifying Therapies That Will Sensitize Tumors to ICI

Another active area of research involves identifying existing therapies that can be used in combination with anti-PD-1 to make tumors more responsive to ICI. Several pathways previously identified as important for tumorigenesis are further being defined as important for immune cell functioning. As our understanding of tumor biology has grown to include the microenvironment, so has our understanding of how targeted therapies can be leveraged to affect the microenvironment. Antibody drug conjugates (ADCs) are another drug class being tested in combination with ICI in TNBC with promising preliminary results. Furthermore, our understanding of how traditional chemotherapies affect the microenvironment has also grown. PTX or nab-PTX are considered standard of care for use in combination with anti-PD-1 in TNBC. The effect that PTX and other traditional chemotherapies may have on immune cell populations is being investigated to maximize potential ICI benefits.

### 6.1. PI3K/AKT Pathway Inhibition

**Figure 3:**
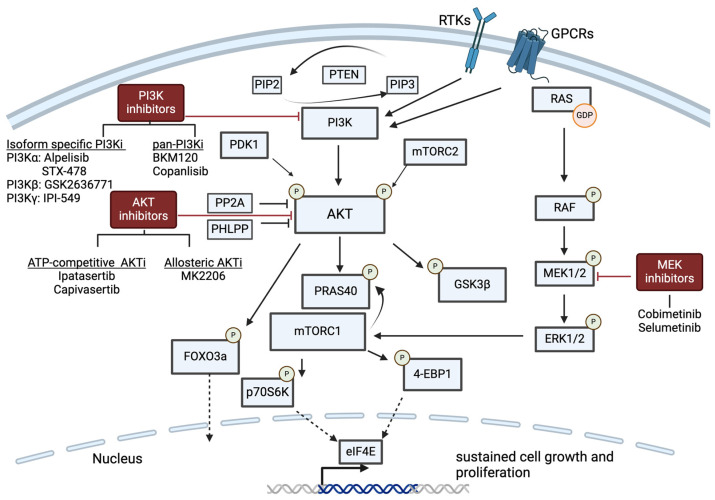
Crosstalk between PI3K/AKT and RAS/MAPK/ERK pathways and the location of action of relevant inhibitors. PI3K, AKT, and MEK inhibitors used in ongoing or completed clinical trials of TNBC are shown in Table 1, Table 2 and Table 3 shown in the diagram. Created with BioRender.com (accessed on 9 May 2024).

The phosphatidylinositol 3-kinase/protein kinase B (PI3K/AKT) pathway is a key signaling pathway with roles in tumor progression as well as immune cell signaling (Figure 3). There are three classes of PI3Ks; however, class I PI3Ks are the most relevant in the context of tumor biology [54]. PI3Ks are made up of a catalytic and a regulatory subunit, and there are four catalytic isoforms: p110*α*, p110β, p110*δ*, and p110*γ*, encoded the PIK3CA, PIK3CB, PIK3CD, and PIK3CG genes, respectively [55]. p110*α* and p110β are expressed ubiquitously in mammalian cells; however, p110*δ*, and p110*γ* are expressed primarily in immune cells, including both lymphoid and myeloid lineage cells [54]. Upon upstream activation of receptor tyrosine kinases (RTKs) or G-protein-coupled receptors (GPCRs), PI3K localizes to the cell membrane, where it phosphorylates phosphatidylinositol-4,5-bisphophate (PIP2), generating phosphatidylinositol-3,4,5-trisphophate (PIP3). PIP3 recruits the Ser/Thr kinase, AKT, to the cellular membrane, where it undergoes a conformational change, allowing AKT to be phosphorylated at S473 by mTORC2 and T308 by PDK1 [54]. Upon phosphorylation, AKT is activated and able to phosphorylate more than 40 downstream substrates, including PRAS40, GSK3β, and FOXO3 [56]. Several phosphatases, including PTEN, PHLPP1/2, PP2A, and SHIP, negatively regulate signaling through this pathway [57,58]. Outputs of PI3K signaling include increased cell proliferation, enhanced metabolism, polarization of macrophages to an M2-protumor phenotype [59], and memory CD8+ T-cell differentiation [60].

There have been widespread efforts to develop pan- as well as isotype-specific PI3K inhibitors, especially for breast cancer, since approximately 50% of all breast cancers have activating alterations in the PI3K/AKT pathway [61,62,63]. In TNBC specifically, about 16% and 11% of tumors exhibit the PIK3CA mutation or loss of the regulatory phosphatase PTEN, respectively [64]. Furthermore, hyperactivation of the PI3K pathway is associated with poor responses to checkpoint inhibitor therapy. In a study of tumors from melanoma patients, loss of PTEN was correlated with decreased intratumoral infiltration of CD8+ T-cells as well as a poor response to anti-PD1 therapy [65]. As a mechanistic follow-up, tumor cell loss of PTEN resulted in less T-cell-mediated killing in co-cultures of human melanoma cell lines and TILs [65]. Furthermore, high pAKT expression in tumor samples predicted poor clinical outcomes in a cohort of ipilimumab-treated melanoma patients [66], and high pAKT-S473 was associated with decreased immune cell infiltrate and decreased IFN*γ* signaling in TNBC patients treated with a triplet therapy consisting of AKT inhibition, PTX, and anti-PD-L1 [67]. Given the recent clinical introduction of ICI to TNBC treatment, the frequency of PI3K pathway mutations in this clinical population, and the immunomodulatory roles of the pathway, there is interest in using PI3K pathway inhibitors to enhance the effects of ICI.

#### 6.1.1. Isoform-Specific PI3K Inhibition 

Several isoform-specific PI3K inhibitors have been explored pre-clinically as well as clinically for breast cancer treatment. Relevant ongoing and completed trials are summarized in Table 1. PI3K*α* and PI3Kβ are PI3K isoforms found in all mammalian cells. Alpelisib is a PI3K*α*-specific inhibitor approved for the treatment of HR+ and HER2− tumors with PIK3CA mutations [68]. It is also being investigated for use on TNBC. Alpelisib is expected to be most effective in the context of PIK3CA-mutated tumors, and there are currently two clinical trials testing alpelisib in combination with nab-PTX in advanced TNBC with either PIK3CA mutation or PTEN loss (NCT04251533 and NCT04216472). While most effective in tumors with PIK3CA mutations, alpelisib has equal affinity for wild-type and mutated PI3K*α*. Inhibiting wild-type PI3K*α* can cause hyperglycemia and insulin resistance, the two major adverse events associated with alpelisib discontinuation [69]. An allosteric, mutant-specific PI3K*α* inhibitor, STX-478, was developed to reduce these off-target metabolic effects. STX-478 showed pre-clinical efficacy and reduced toxicity in ER+ HER2− xenograft models [70], and a phase 1 clinical trial of STX-478 for the treatment of PIK3CA mutant advanced tumors, including breast cancer, is currently recruiting (NCT05768139). Pre-clinical studies have not identified major immunomodulatory mechanisms of PI3K*α* inhibition. Alpelisib, in combination with PTX, failed to increase the efficacy of ICI in the C57BL/6J PyMT mouse orthotopic tumor model of TNBC, and there was no effect on T-cell recruitment or activation [71]. Furthermore, genetic inactivation of PIK3C*α* in a PTEN/P53 null mouse model of breast cancer did not significantly alter the immune cell profile of the tumors [72]. Consequently, PI3K*α*i may not be a strong candidate for improving the efficacy of ICI. While genetic inactivation of PIK3C*α* did not alter immune cell signaling, work from the same group established PI3Kβ as a key player in promoting an immune evasion phenotype in breast tumors. Genetic inactivation of PI3Kβ drastically altered the TME by increasing the recruitment of activated CD8+ T-cells, M1-like macrophages, and dendritic cells, while pharmacological inhibition of PI3Kβ synergized with anti-PD1 to cause complete tumor regression in 3 of 6 mice [72]. Currently, there are no FDA-approved PI3Kβ-specific inhibitors; however, studies are underway investigating PI3Kβi for solid tumors with PTEN loss (NCT01458067). The phase I/IIa trial of the PI3Kβ inhibitor, GSK2636771, did include 6 TNBC patients, representing 9% of the total study population [73]. GSK2636771 is also being tested in combination with anti-PD1 for metastatic melanoma patients with PTEN loss (NCT03131908). Given the promising pre-clinical studies, there may be a benefit to testing PI3Kβ inhibitors in combination with anti-PD1 in TNBC populations. 

PI3K*δ* and PI3K*γ* are PI3K isoforms expressed specifically in immune cells. PI3K*δ* is primarily expressed by B and T cells. Several PI3K*δ*-specific inhibitors are FDA-approved for hematological malignancies such as chronic lymphocytic leukemia and follicular lymphoma, but they hold less promise as treatments for solid tumors where there is conflicting evidence as to the effect of PI3K*δ* inhibition on T-cell function [74]. Genetic inactivation of PI3K*δ* in the 4T1 mouse TNBC model reduced tumor growth and reduced regulatory T-cell functions in peripheral tissues as well as in tumor-draining lymph nodes [74]. Despite the positive effects on regulatory T-cells, there are proposed negative effects on cytotoxic CD8+ T-cell populations that may ultimately result in an immunosuppressive TME. PI3K*δ* inhibition reduced CD8+ T-cell-mediated cytotoxicity against tumor cells in vitro and was found to antagonize rather than enhance ICI in the MC38-OVA mouse colon cancer model [75]. Another group compared the effects of PI3K*δ* inhibition on naïve versus effector cytotoxic CD8+ T-cells in vitro and described mixed effects on different T-cell functions. Overall, the transcriptional profile of naïve CD8+ T-cells was affected more by PI3K*δ* inhibition than effector cells [76]. They observed the downregulation of a family of genes broadly labeled ‘immune response molecules’ and the downregulation of genes, such as FOXO1, involved in T-cell differentiation. Conversely, PI3K*δ* inhibition in effector cytotoxic CD8+ T-cells increased expression of genes related to T-cell trafficking to and from lymphoid tissue, which could enhance T-cell trafficking to tumors [76]. This study suggests that PI3K*δ* inhibition may not negatively affect an existing CD8+ anti-tumor response, but it may hinder the conversion of naïve CD8+ T-cells to effector CD8+ T-cells. While inhibiting PI3K*δ* may not enhance ICI in solid tumors, inhibiting PI3K*γ* is a promising immunomodulatory strategy for solid tumors, including breast cancer. PI3K*γ* has been described as a molecular switch that modulates macrophage polarization [77]. Since breast cancers are macrophage-rich tumors, a therapy that relieves macrophage immunosuppression may be effective and combine well with ICI. In the 4T1 mouse model of TNBC, the gamma-specific inhibitor, IPI-549, increased the M1/M2 ratio, enhanced the effector T-cell population, and sensitized tumors to ICI [78]. Furthermore, MMTV-PyMT tumors grew slower in mice lacking PI3K*γ* (PI3K*γ*−/−) and exhibited enhanced intratumoral infiltration of CD8+ T-cells compared to tumors in PI3K*γ*-wild-type mice [79]. While no PI3K*γ* inhibitors are currently FDA-approved, trials are underway. The MARIO-1 phase I/Ib trial of IPI-549 combined with anti-PD1 for TNBC treatment showed acceptable safety profiles [80], and the phase II MARIO-3 trial is currently underway. Interim analysis from MARIO-3 suggests an improvement in one-year PFS when compared to data from the IMpassion130 trial (anti-PD-L1+PTX) [81].

**Table 1:** Ongoing and completed clinical trials of isoform-specific PI3K inhibitors in BC.

Identifier	Phase	Combination	Drug Names	Indications	Results	Ref
NCT04216472	II	PI3K*α*i + Chemo	Alpelisib + nab-PTX	Anthracycline-resistant TNBC with PIK3CA mutation or PTEN loss	Active, not recruiting	n/a
NCT04251533	III	PI3K*α*i + Chemo	Alpelisib + nab-PTX	Advanced stage TNBC with PIK3CA mutation or PTEN loss	Active, not recruiting	n/a
NCT05768139	I/II	PI3K*α*i (mutation specific)	STX-478	Advanced solid tumors with PIK3CA mutation	Recruiting	n/a
NCT01458067	I/IIa	PI3Kβi	GSK2636771	Solid tumors with PTEN loss	Acceptable safety/toxicity profile	[73]
9 + NCT04439188 (MATCH-Sub-protocol P)	II	PI3Kβi	GSK2636771	Cancers with PTEN loss	Active, not recruiting	n/a
NCT02637531 (MARIO-1)	I/Ib	PI3K*γ*i +/− anti-PD1	IPI-549 (eganelisib) +/− Nivolumab	TNBC	Acceptable safety/toxicity profile	[80]
NCT03961698 (MARIO-3)	II	PI3K*γ*i + anti-PD-L1 + Chemo	IPI-549 (eganelisib) + atezolizumab + nab-PTX	Locally advanced unresectable or metastatic TNBC	Interim analysis: ORR 55.3%	[81]

#### 6.1.2. Pan-PI3K Inhibition and AKT Inhibition

One limitation of isotype-specific inhibitors is that the upregulation of non-inhibited isoforms can cause therapy resistance. Several pan-PI3K inhibitors were developed and have been tested pre-clinically and clinically. The strategy of pan-PI3K inhibition circumvents the problem of compensatory upregulation of non-inhibited isoforms, however, often at the cost of greater toxicity. For example, the pan-PI3K inhibitor BKM120 was previously shown to enhance anti-PD1 efficacy in the C57BL/6J PyMT orthotopic tumor model by increasing the recruitment of CD8+ T-cells [79]. Clinically, however, BKM120 failed to progress after a phase II trial revealed unacceptable toxicity profiles [82]. Copanlisib, a pan-PI3K inhibitor, and gedatolisib, a PI3K/mTOR inhibitor, also synergized with ICI in the PyMT orthotopic mouse tumor model [71] and are being investigated clinically (NCT04345913; NCT01920061) for advanced-stage TNBC. Like concerns previously discussed with PI3K*δ* inhibition, a pan-PI3K inhibition approach may also suppress CD8+ T-cell populations [83]. Using the pan-PI3K inhibitor, KTC1101, one group demonstrated that they could overcome this problem by altering the dosing and treatment schedule [84]. Pre-treatment with anti-PD-1 followed by intermittent rather than continuous dosing of KTC1101 optimally inhibited regulatory T-cells while maintaining CD8+ T-cells [84].

An alternative, less toxic approach to inhibiting the PI3K pathway involves inhibiting AKT, the Ser/Thr kinase just downstream of PI3K. Two classes of AKT inhibitors—ATP competitive and allosteric inhibitors—are the subjects of investigation for BC treatment. ATP-competitive inhibitors of AKT include ipatasertib (IPAT) and capivasertib (CAPI), both of which show an affinity for all three AKT isoforms (IPAT IC50 AKT1/2/3 = 5 nM, 18 nM, 8 nM [56]; CAPI IC50 AKT1/2/3 = 3 nM, 7 nM, 7 nM [85]). IPAT and CAPI bind to the ATP-binding pocket of AKT, locking the protein in a conformation inaccessible to phosphatase activity. Consequently, AKT remains phosphorylated but is not able to phosphorylate downstream targets. Experimentally, protein levels of pAKT are not expected to decrease in response to treatment and may even increase [56]. Such AKT inhibitors are thought to have reduced toxicity compared to pan-PI3K inhibitors because, by preferentially binding to pAKT, they are selective for activated AKT. IPAT and CAPI both exhibited acceptable safety profiles in early-phase clinical trials [86,87]. However, measures of efficacy have been mixed, pointing to a need for better biomarkers to refine patient selection criteria. For example, in the phase II LOTUS trial of IPAT plus PTX for metastatic TNBC, there was a trend toward increased overall survival (OS) with the combination treatment for PTEN low and PIK3CA/AKT1/PTEN-altered subgroups (25.8 vs. 22.1 mo) [88], but in the subsequent phase III IPATunity130 trial, there was no improvement in progression-free survival [89].

Allosteric inhibitors bind inactive AKT at sites other than the ATP-binding pocket to stabilize the PH-kinase domain [90]. An example of a well-studied allosteric AKT inhibitor is MK-2206. Unlike ATP-competitive inhibitors, AKT is not able to be phosphorylated when bound to an inhibitor, and decreases in protein levels of pAKT will be observed in response to treatment [56]. MK-2206 may have important immunomodulatory functions. Patients with HR+/HER2− breast cancer were treated with MK-2206, and formalin-fixed paraffin-embedded (FFPE) slides from diagnostic biopsies were compared to slides from post-treatment surgical samples (NCT013195390). Immunofluorescence staining showed an increase in the density of CD8+ T-cells in post-treatment samples, and nanostring gene expression analysis showed an increase in interferon signaling and pro-apoptotic genes in response to MK-2206 [91]. MK-2206 is also included as a treatment arm in the I-SPY2 platform trial, which is testing several agents as a neoadjuvant for high-risk breast cancer [92]. The different mechanisms of action of allosteric versus ATP-competitive inhibitors are important when considering biomarkers of response and mechanisms of resistance. For example, in the I-SPY2 trial, low pAKT was associated with response in the TNBC cohort [93], but the opposite may be true for response to ATP-competitive inhibition [94]. Genetic mutation in AKT1 is associated with resistance to MK-2206 but not IPAT, and compensatory signaling through PIM-1 is associated with resistance to IPAT but not MK-2206 [95]. While acting through different mechanisms, both types of AKT inhibitors may have a place in TNBC treatment if consistent biomarkers of response can be established to guide patient selection criteria. Furthermore, there is interest in combining AKT inhibitors with immune checkpoint inhibitors, including the ongoing clinical trials NCT04177108, NCT03424005, and NCT03742102 (Table 2).

**Table 2:** Ongoing and completed clinical trials of pan-PI3K and AKT inhibitors in BC.

Identifier	Phase	Combination	Drug Names	Indications	Results	Ref
NCT01790932	II	pan-PI3Ki	BKM120 (buparlisib)	Metastatic TNBC	No OR	[82]
NCT04345913	I/II	pan-PI3Ki + Chemo	Copanlisib + Eribulin	Advanced stage TNBC	Active, not recruiting	n/a
NCT01920061	I/II	pan-PI3Ki + Chemo	Copanlisib+ Eribulin	Advanced-stage TNBC	Active, not recruiting	n/a
NCT01090960	I	AKTi	IPAT	Metastatic TNBC	Acceptable safety/toxicity profile	[86]
LOTUS	II	AKTi + Chemo	IPAT + PTX	Metastatic TNBC	Trending increase OS for PTEN low and PIK3CA/AKT1/PTEN-altered subgroups (25.8 vs. 22.1 mo)	[88]
FAIRLANE	II	AKTi + Chemo	IPAT + PTX	Early-stage TNBC	No increase in pCR	[96]
IPATunity130	III	AKTi + Chemo	IPAT + PTX	Locally advanced unresectable or metastatic TNBC	No improvement in PFS	[89]
PAKT	II	AKTi + Chemo	CAPI + PTX	Metastatic TNBC	PFS (5.9 vs. 4.2 months) OS (19.1 vs. 12.6 months)	[87]
CAPItello-290	III	AKTi + Chemo	CAPI + PTX	Metastatic TNBC	Active, not recruiting	[97]
NCT03742102 (BEGONIA)	Ib/II	AKTi + anti-PD-L1 + Chemo	CAPI + Durvalumab+PTX	Metastatic PD-L1+ TNBC	Interim analysis: no change in ORR	[98]
NCT03800836 (CO40151)	Ib	AKTi + anti-PD-L1 + Chemo	IPAT + Atezolizumab + PTX or nab-PTX	Locally advanced or metastatic TNBC	Acceptable safety/toxicity profile: 73% ORR	[99]
NCT03424005 (Morpheus-panBC)	Ib/II	AKTi + anti-PD-L1	IPAT + Atezolizumab	Locally advanced unresectable or metastatic TNBC	Recruiting	n/a
NCT04177108 (IPATunity170)	III	AKTi + anti-PD-L1 + Chemo	IPAT + Atezolizumab + PTX	Locally advanced unresectable or metastatic TNBC	No improvement in PFS or ORR	[67]
NCT01263145	Ib	AKTi + Chemo	MK-2206 + PTX	Metastatic breast cancer	Acceptable safety/toxicity profile	[100]
NCT01277757	II	AKTi	MK-2206	Advanced breast cancer with PIK3CA mutation, AKT mutation, or PTEN loss	No improvement in PFS	[101]

### 6.2. RAS/MAPK/ERK Pathway Inhibition

The Ras/MAPK/ERK signaling pathway is another key oncogenic pathway with immunomodulatory properties that is implicated in resistance to immunotherapy. For example, genetic alterations in the Ras/MAPK pathway were associated with reduced levels of TILs in post-neoadjuvant chemotherapy (post-NAC) TNBC biopsies [102], and there is a strong association between TILs and pCR [7]. Rat sarcoma virus (RAS) proteins are G-proteins that exist in an active conformation when bound to guanosine diphosphate (GDP) and an inactive conformation when bound to guanosine triphosphate (GTP). HRAS, NRAS, KRAS4A, and KRAS4B are four isoforms of RAS [103]. Activation of RAS through upstream GPCRs leads to subsequent activation of the MAPK/ERK and PI3K/AKT signaling cascades. There is substantial crosstalk between the PI3K/AKT and MAPK/ERK signaling pathways, which converge on the initiation and regulation of eIF4E cap-dependent translation [104] (Figure 3). The MAPK pathway can activate mTORC1, bypassing the effects of AKT inhibition to phosphorylate 4E-BP1 and allowing the downstream initiation of eIF4E cap-dependent translation [104]. Consequently, MAPK mutations have been implicated in resistance to PI3K/AKT inhibitors [86,94]. RAS mutations are very rare in human BC; however, mutation or enhanced signaling through upstream receptor inputs can lead to signaling amplification [105]. RAS is frequently considered the “undruggable oncogene” because of its poor binding pockets, but inhibition of downstream targets such as mitogen-activation protein kinase (MEK) can be achieved with existing small-molecule inhibitors. Four MEK inhibitors are currently FDA-approved: selumetinib for neurofibromatosis I, trametinib for anaplastic thyroid cancer, non-small cell lung cancer, and metastatic melanoma, binimetinib for metastatic melanoma, and cobimetinib for metastatic melanoma [106]. Preclinical work suggests that MEK inhibitors may be effective in BC as well. For example, in mouse xenograft models of TNBC, selumetinib significantly reduced lung metastasis [107]. Given the substantial crosstalk between the two pathways, there has been interest in combining AKT and MEK inhibitors; however, when tested clinically, there is substantial toxicity and limited efficacy [108].

There are conflicting reports, however, as to whether MEK inhibition hinders or synergizes with ICI therapy. For example, in the CT26 mouse colon cancer model, MEK inhibition synergized with anti-PD-L1 therapy to reduce tumor growth while exerting conflicting, site-specific effects on T-cells [109]. MEKi suppressed anti-tumor T-cell priming in the lymph node but protected CD8+ T-cells from apoptosis once in the tumor [109]. In murine models of TNBC, MEKi reduced infiltration of both antigen-specific and non-antigen-specific CD8+ T-cells [110]. Furthermore, analysis of peripheral blood samples from metastatic bile duct cancer patients receiving atezolizumab alone or in combination with cobimetinib showed that the addition of cobimetinib significantly reduced the proportion of activated CD8+ T-cells in circulation [111]. This suggests that MEKi may synergize with ICI in a tumor with a pre-existing CD8 infiltrate but may hinder the recruitment of activated CD8+ T-cells in the context of an immune cold tumor. Other studies, however, suggest that despite potential negative effects on T-cell effector functions, MEKi may synergize with ICI through increasing tumor cell immunogenicity. In mouse models of BC, MEK inhibition activated STAT signaling, increasing MHC-1 and PD-L1 expression in tumor cells [112]. Similarly, in a model of intrahepatic cholangiocarcinoma, MEKi re-sensitized tumors to anti-PD-1 therapy through upregulation of MHC-1 [113]. Given the conflicting reports of MEKi in combination with ICI, further work is needed to determine optimal dosing strategies to minimize potential negative effects on T-cell effector functions while maximizing effects on tumor cell immunogenicity. While no results have been published, there are two registered clinical trials that examine MEKi in combination with anti-PD-L1 in BC (NCT03801369, NCT03202316) (Table 3).

**Table 3:** Ongoing and completed clinical trials of MEK inhibitors in BC.

Identifier	Phase	Combination	Drug Names	Indications	Results	Ref
NCT01562275	Ib	AKTi + MEKi	Ipatasertib + Cobimetinib	Locally advanced or metastatic solid tumors	Limited tolerability and efficacy	[108]
NCT03202316	II	Anti-PD-L1 + MEKi + Chemo	Atezolizumab + Cobimetinib + Eribulin	Chemotherapy-resistant metastatic inflammation breast cancer	Active, not recruiting	n/a
NCT03801369	II	PARPi + anti-PD-LI or MEKi or AKTi	Olaparib + Durvalumab, or Selumetinib, or Capivasertib	Metastatic TNBC	Recruiting	n/a

### 6.3. High Throughput Screening to Identify Targeted Therapies

Beyond the PI3K/AKT and RAS/MAPK/ERK inhibitors discussed above, there exist thousands of inhibitors against key tumorigenic pathways. High-throughput screening approaches are being used to identify the immunomodulatory functions of such existing compounds. Repurposing compounds for new indications is often more cost- and time-effective than developing a novel compound into a clinical candidate, thereby shortening the time it takes for therapies to safely be available to patients. While the complete complexity of the in vivo TME cannot be recreated in culture, platforms are being developed to examine specific interactions, such as T-cell-mediated cytotoxicity of tumor cells. For example, Lizotte et al. utilized co-cultures of ID8-OVA ovarian cancer cells and CD8+ OT-1 splenic T-cells to screen a compound library of kinase inhibitors for evidence of enhanced T-cell-mediated cytotoxicity [114]. They identified the EGFR inhibitor, erlotinib, as a top hit and further confirmed in a mouse model of ovarian cancer that EGFR inhibition synergized with anti-PD-1 [114]. Another group utilized a similar strategy to screen 850 bioactive compounds for evidence of enhanced T-cell killing of tumor cells in human melanoma cell lines, identifying HSP90 inhibition as an immunomodulatory strategy [115]. Another platform involves co-culturing tumor cells with irradiated tumor cells and TILs rather than splenocytes [116]. The irradiated tumor cells enhance antigen cross-presentation, and TILs are more representative of the TME than are splenocytes.

Three-dimensional culture models more closely recapitulate the in vivo TME, and there are several methodologies being developed to adapt high-throughput screening approaches to 3D co-cultures or patient derived organoids (PDOs). One group utilized E0771-OVA-expressing mouse TNBC organoids co-cultured with OT-1 CD8+ splenic T-cells to screen a library of epigenetic modulators, identifying three candidates with the potential to synergize with anti-PD-1 [117]. Another group screened a herbal medicine library against co-cultures of OVA-expressing colorectal cancer spheroids and OT-1 CD8+ T-cells, identifying atractylenolide I (ATT-1) as a compound that improves T-cell cytotoxicity through enhanced antigen presentation [118]. High-throughput immune-oncology platforms in 2D and 3D cultures have led to the identification of candidate drugs that may combine with anti-PD1 therapy in breast and other cancers.

### 6.4. Antibody–Drug Conjugates

Combining ADCs with ICI is another treatment strategy being investigated for TNBC. ADCs are composed of a monoclonal antibody linked to a cytotoxic payload, which allows for increased tumor targeting [119]. Proposed mechanisms of ADCs enhancing ICI effects include enhancement of tumor cell MHCI expression and antibody-dependent cell cytotoxicity (ADCC), stimulating an adaptive immune response [120]. Trastuzumab deruxtecan (T-Dxd) is an ADC that is FDA-approved for metastatic HER-2 low BC, including HER-2 low TNBC, as a result of the DESTINY-Breast04 trial [5]. The antibody component, trastuzumab, targets HER-2, and the payload, deruxtecan, is a topoisomerase I inhibitor. T-Dxd, in combination with anti-PD-L1, is being investigated as a treatment arm of the phase Ib/II BEGONIA trial for metastatic TNBC (NCT03742102). Preliminary measures of toxicity and efficacy are promising, with no dose-limiting toxicities reported in the interim analysis [121]. Sacituzumab govitecan is another ADC being investigated in combination with anti-PD-1 in TNBC. Similar to T-Dxd, the payload is a topoisomerase I inhibitor. The antibody component, sacituzumab, targets trophoblast cell-surface antigen 2 (Trop-2), which is highly expressed in breast cancer [122]. This ADC is FDA-approved for metastatic TNBC as a result of the phase III ASCENT trial [122]. A phase II clinical trial of sacituzumab govitecan in combination with anti-PD-1 for the treatment of metastatic TNBC is currently underway (NCT04468061). Clinical trials are also planned to test a similar Trop-2-targeted ADC, sacituzumab tirumotecan, in combination with anti-PD-1 in patients with TNBC (NCT06393374) as well as in patients with metastatic ER+/HER2*−* BC (NCT06312176).

### 6.5. Chemotherapies

Traditional, cytotoxic chemotherapies were first used clinically in the 1900s with little understanding of their mechanism of action, and pre-clinical studies relied heavily on immunocompromised mouse models [123]. With the recognition that immune cells play vital roles in the emergence and progression of a tumor, as well as the clinical introduction of immune checkpoint inhibitors, there is interest in the potential immunomodulatory mechanisms of the actions of these standard chemotherapies. Identifying which chemotherapies, and at what doses, best synergize with immunotherapy is an active area of research.

Broadly speaking, standard, cytotoxic chemotherapy agents act by interfering with the replication of rapidly dividing cells. For example, PTX stabilizes microtubules during cell division, causing mitotic cell arrest and apoptosis [124], etoposide inhibits topoisomerase II [125], and platinum-based agents, such as cisplatin, crosslink with DNA to cause mitotic arrest [126]. It is now recognized that this cytotoxicity can also be immunostimulatory. Immunogenic cell death (ICD) describes a form of regulated cell death that stimulates a response from the adaptive immune system [127]. ICD can involve the secretion of ATP, the translocation of calreticulin and heat shock family proteins to the cell surface, and the release of high mobility group box 1 (HMGB1) [127]. Several chemotherapies have been described as ICD inducers in pre-clinical studies, including PTX, bleomycin, anthracyclines, and cisplatin, just to name a few [128].

While cytotoxic chemotherapy can cause ICD, it can also be immunosuppressive, especially when used in a dose-dense treatment regimen. The K522 regimen for TNBC patients is an example of one such intensive strategy that includes pembrolizumab plus PTX, carboplatin, doxorubicin, and cyclophosphamide [34]. Ongoing work is investigating whether the same benefits can be achieved while removing doxorubicin as a de-escalation strategy to minimize the amount of chemotherapy necessary [30]. PTX is routinely used in TNBC treatment; however, patients often require steroid pre-treatment because PTX is dissolved in a polyethylated castor oil that can induce hypersensitivity reactions [129]. Nab-PTX, however, does not require steroid pretreatment. In the GeparSepto phase III trial of nab-PTX versus solvent-based PTX in metastatic BC patients, more patients in the nab-PTX group achieved a pCR than in the solvent-based PTX group, although peripheral neuropathy was more frequently reported in the nab-PTX group [130]. However, another group performed an observational study using data from the electronic health records of metastatic BC patients and found no overall differences in efficacy between PTX and nab-PTX [131]. PTX and nab-PTX have both been tested in combination with anti-PD-1 clinically in TNBC. The data are not definitive, but there is some concern that the immunosuppressive steroid pre-treatment may make PTX less effective than nab-PTX in combination with ICI [132].

## 7. Identifying New Immunotherapy Strategies for TNBC beyond Anti-PD-1/PD-L1

While anti-PD-1 or anti-PD-L1 therapies have revolutionized treatment for certain cancer types, such as melanoma, these two antibodies simply may not be the most effective immunotherapy strategies for BC. In this section, we will describe efforts to identify new immunotherapy strategies for BC, including targeting alternative checkpoint proteins and non-checkpoint immunotherapy strategies.

### 7.1. CTLA-4

PD-1 blockade is currently the only immune checkpoint inhibitor approved for use in BC, while CTLA-4 blockade is approved for use in combination with anti-PD1 or anti-PD-L1 in other cancer indications such as melanoma [133]. Toxicity is a major concern with adding CTLA-4 blockade to PD1/PD-L1 blockade, although there is evidence to suggest that anti-CTLA-4 may be effective in TNBC. In a pilot study of durvalumab (anti-PD-L1) and tremelimumab (anti-CTLA-4) for metastatic breast cancer, only three of 18 patients responded to therapy. However, all three responding patients had TNBC rather than HR+ disease [134]. No grade 4 or 5 adverse events occurred with combination treatment. In the DART trial (NCT02834013) examining the combination of anti-PD1 and anti-CTLA-4 in rare tumors, three patients with metaplastic breast cancer, which is similar to claudin-low and mesenchymal TNBC subtypes, exhibited ongoing responses of almost three years at last follow-up [135]. Compared to monotherapy of either agent, combinations of anti-PD1 and anti-CTLA-4 work through distinct cellular mechanisms [136,137,138]. Despite the risk of increased toxicity, there may be benefit in using anti-CTLA-4 in cases of advanced TNBC, and several ongoing clinical trials are assessing the benefit of anti-CTLA-4 therapy in BC (NCT03132467, NCT03982173, NCT037899110, NCT03518606, NCT03058289, and NCT02536794). Bispecific antibodies are also being developed to minimize the toxicity of combination checkpoint inhibitor therapy while maintaining efficacy. One such bispecific, MEDI5752, preferentially inhibits CTLA-4 on PD-1+ cells, reducing peripheral toxicity and improving tumor targeting [139]. Promising early clinical results showed improvements in a patient with renal clear cell carcinoma and gastric adenocarcinoma (NCT03530397).

### 7.2. Targeting Alternative Checkpoints

PD-1 and CTLA-4 are just two negative regulatory mechanisms in place to prevent autoimmunity in a homeostatic setting. A plethora of other checkpoint proteins and mechanisms are in place that could potentially be targeted therapeutically. To better understand the checkpoint expression profile in BC, one group analyzed the mRNA expression of 50 immune checkpoint genes in normal breast and breast cancer tissue samples from the Cancer Genome Atlas (TCGA) [140]. TNBC samples exhibited increased expression of CTLA-4, PD-1, lymphocyte activation gene-3 (LAG-3), T cell immunoreceptor with immunoglobulin and tyrosine-base inhibitory motif domain (TIGIT), and indoleamine 2,3-dioxygenase 1 (IDO1). Furthermore, high expression of CTLA-4 or TIGIT correlated with favorable clinical outcomes regardless of BC subtype [140]. Therapeutic antibodies are being developed against several of these checkpoint proteins. An anti-LAG-3 antibody, relatlimab, is currently FDA-approved in combination with nivolumab (anti-PD-1) for advanced melanoma patients based on results from the RELATIVITY-047 trial [141]. A soluble LAG-3 protein that acts as an MHCII agonist is also under development, and a clinical trial is currently recruiting to test this in combination with standard-of-care chemotherapy in metastatic breast cancer patients (NCT05747794). An anti-TIGIT monoclonal antibody, ociperlimab, was found to be well-tolerated and to have preliminary anti-tumor activity in combination with anti-PD-1 in a phase I dose escalation study for patients with advanced solid tumors [142]. A phase II trial of ociperlimab in combination with anti-PD-1 plus chemotherapy as a first-line treatment for patients with advanced TNBC was registered (NCT05809895) but shortly withdrawn, with the sponsoring company citing business decisions rather than new safety concerns with ociperlimab. Unlike LAG-3 and TIGIT, which are receptors expressed on the surface of certain immune cells, IDO1 is an enzyme produced by myeloid lineage cells that can suppress the activity of cytotoxic cells such as T-cells and NK cells. Several small-molecule inhibitors against IDO1 have been developed and tested preclinically and clinically. For example, the IDO1 inhibitor NLG919 synergized with doxycycline to reduce tumor growth in the 4T1 TNBC mouse tumor model [143]. IDO1 clinical trials have been largely negative, however, and no IDO1 inhibitors are currently FDA-approved [144,145]. A phase Ia/Ib study of the IDO1 inhibitor, LY3381916, in combination with anti-PD-1 for solid tumors, including TNBC, was initiated but terminated due to business decisions (NCT03343613).

### 7.3. Novel Immunotherapy Targets

Beyond the aforementioned checkpoint proteins, several screening strategies are being used to identify new immunotherapy targets. For example, an in vivo CRISPR screen identified the RNA helicase, Dhx37, as a modulator of CD8 T cell activation and cytotoxicity in the E0771-OVA model of TNBC [146]. Another study identified Lgals2, the gene encoding the galectin-2, glycan-binding protein, in an in vivo CRISPR screen in the mouse TNBC 4T1 model to identify genes involved in immune escape [147]. Furthermore, antibody blockade of LGALS2 enhanced anti-tumor immune responses in vivo in the 4T1 model. A similar in vivo CRISPR screen in the murine melanoma B16 model identified the protein tyrosine phosphatase, PTPN2, as a potential new immunotherapy target [148]. Such CRISPR screening methodologies hold promise for identifying immunotherapy targets, but a further challenge will be developing and testing therapeutic antibodies against the proposed targets.

### 7.4. Myeloid Based Therapies

Anti-PD-1 acts to delay T-cell exhaustion; however, there are numerous other cell types in the TME that can be targeted therapeutically. ICI is most effective in TIL-rich tumors; however, myeloid cells can make up approximately 50% of the cells in the breast TME [149,150]. Myeloid-based immunotherapies may, therefore, be more effective than T-cell-based immunotherapies in TNBC because they can take advantage of a dense, pre-existing cell population. Myeloid cells, particularly macrophages, exist along a spectrum of phenotypes ranging from pro-tumor (M2) to anti-tumor (M1), and the M1/M2 ratio varies between tumors and in response to treatments [151]. Work is being conducted to understand how current therapies affect M1 versus M2 polarization, with the goal of enhancing M1 polarization. Novel macrophage-targeted therapies include the expression of chimeric antigen receptors (CARs) [152], macrophage-targeted nanoparticles [153], and macrophages as drug carriers [154], just to name a few. We will briefly touch on recent advances in the development of chimeric antigen receptor macrophages (CAR-Ms). CAR-Ms designed to express vascular endothelial growth factor receptor-2 (VEGFR2) inhibited tumor progression in the 4T1 murine model of TNBC [155]. Another group engineered CAR-Ms to activate signaling through CD147 upon engagement with HER2 [156]. CD147 signaling activates matrix metalloproteinases (MMPs), reducing collagen deposition, reducing tumor growth, and enhancing T-cell infiltration in the HER2-4T1 murine model of TNBC [156]. A phase I clinical trial is active, but not yet recruiting, to investigate anti-HER2 CAR-Ms in HER2 overexpressing solid tumors, including BC (NCT04660929). If successful, such a strategy could be applied to HER-low TNBCs.

## 8. Conclusions and Future Directions

While the introduction of anti-PD-1 to the clinical treatment of TNBC represents a major advance, few patients achieve a durable response to therapy, necessitating the development of strategies to improve response rates. In this review, we highlight three main approaches for improving immunotherapy response rates in TNBC: improving patient selection, identifying existing therapies that will enhance ICI, and identifying new immunotherapy strategies beyond anti-PD-1/anti-PD-L1 (Figure 1). The development and refinement of biomarkers to predict which patients will respond to anti-PD-1 will be essential to guiding treatment decisions to minimize potential adverse effects and maximize the potential benefit of ICI. scRNA-seq and spatial transcriptomics of patient samples are being used to define signatures of response and resistance, and these may be adapted to clinical trial designs and analyses [47,53,157]. A composite biomarker incorporating tumor and immune cell signatures will likely be the most successful approach. Upon identification and validation of such a biomarker, additional challenges will include standardizing diagnostic assays across clinical sites and ensuring that relevant clinical populations have access to testing. Identifying existing therapies, including chemotherapies, ADCs, or targeted therapies that may synergize with ICI, is another active area of research. The K522 regimen, consisting of pembrolizumab, paclitaxel, carboplatin, doxorubicin, and cyclophosphamide, is the standard of care for most stage II and III TNBC patients [34]. While chemotherapy can be immunostimulatory, dose-dense regiments such as the K522 protocol can be immunosuppressive, and there is work being conducted to determine optimal chemotherapy partners to use with anti-PD-1. ADCs are promising candidates for use in combination with ICI, and several clinical trials are underway exploring this combination in TNBC. Many targeted therapies are also being investigated for their ability to synergize with anti-PD-1. In this review, we focused on recent work exploring PI3K/AKT and MAPK pathway inhibitors as combinatorial strategies. We discuss the benefits and limitations of pan- as well as isotype-specific PI3K inhibitors and allosteric as well as ATP-competitive AKT inhibitors. While clinical trials of PI3K or AKT inhibition in combination with anti-PD-1 for TNBC have largely been negative, there is evidence to suggest that the biomarker selection patient population may still benefit [67,94]. Pre-clinical studies of MAPK pathway inhibition using MEK inhibitors suggest synergy with anti-PD-1 through upregulating MHCI expression on tumor cells [112], but reports of effects on CD8+ T-cells have been mixed [109,110]. Nevertheless, there are two active clinical trials investigating MEK inhibition in combination with anti-PD-L1 in BC (Table 3). The last approach explored in the present review is perhaps the most promising—identifying novel immunotherapy approaches beyond anti-PD-1/PD-L1. It is possible that targeting the PD-1/PD-L1 axis is simply not the most effective approach in TNBC. Targeting alternative checkpoints, identifying novel immunotherapy targets, or developing myeloid or other cell-based therapies are all active areas of research that may hold the key to the next breakthrough in TNBC treatment.
